# AI-Driven Internet of Things (IoT) dataset for remote health monitoring and fall detection in elderly people

**DOI:** 10.1016/j.dib.2026.112641

**Published:** 2026-03-03

**Authors:** Md. Reazul Islam, Md. Owafeeuzzaman Patwary, S M Ashiqul Islam

**Affiliations:** a Department of Computer Science, University at Albany, Albany, NY, USA; b Department of Computer Science, American International University-Bangladesh, Dhaka 1229, Bangladesh; c Department of Epidemiology & Biostatistics, University at Albany, Albany, NY, USA

**Keywords:** Elderly care, Internet of Things (IoT), Machine learning, Remote health monitoring, Fall detection, Sensor data, MQTT, Simulated dataset

## Abstract

Many older adults live with one or more chronic conditions that require ongoing monitoring. At the same time, the aging population continues to grow, increasing interest in remote health management solutions, particularly for seniors who live alone and may face delayed assistance during medical emergencies. In this data article, we present a comprehensive simulated dataset designed to represent IoT-based remote health monitoring and fall detection scenarios in older adults. The dataset incorporates multimodal sensor data capturing physiological signals (heart rate, blood oxygen saturation (SpO₂), and body temperature) and motion-related measurements (three-axis acceleration and rotation). The dataset consists of raw inertial data, derived magnitudes, heart rate, and heart rate variability values, along with their timestamps from the sensors. It contains 2D and 3D human skeletons, and each record is labeled by a health condition (Normal, Hypertension, Hypotension, Fever, Hypoxia, Fall) with binary feature variables representing a label for fall detection and a label for health risk, respectively. This dataset serves as a promising benchmark for training and testing machine learning methods, including support vector machines (SVM), random forests, gradient boosting, and logistic regression, to automatically classify health status and critical event detection. The dataset is intended to support benchmarking and comparative evaluation of machine learning methods for health status and critical event classification.

Specifications Table

**Table:** 

Subject	Elderly Health Monitoring and Fall Detection Dataset
Specific Subject Area	Remote Health Monitoring, IoT-based Elderly Care
Data Format	CSV (multimodal physiological and motion data)
Type of Data	Simulated physiological and kinematic sensor data
Data Collection	Data generated through structured simulation of wearable IoT sensors (heart rate, SpO₂, temperature, accelerometer, gyroscope, GPS)
Data Source Location	N/A (simulated data; no physical location)
Data Accessibility	Repository name: Mendeley Data Data identification number: DOI: 10.17632/tf56mbyg69.1 Direct URL to data: islam, reazul (2025), “Remote Health Monitoring and Fall Detection in Elderly People”, Mendeley Data, V1, doi: 10.17632/tf56mbyg69.1

## 1 Value of the Data

• This dataset provides a pre-labelled training and testing resource for machine learning algorithms in remote elderly care, where real-world data are scarce and expensive.
• The inclusion of both physiological (heart rate, SpO2, temperature) and kinematic (accelerometer, gyroscope) data in the same dataset allows the generation of fusion models that link changes in vital signs with falls or activities.
• There is an opportunity to use these data to train multi-class classification models that not only detect falls but also specific abnormal states, such as hypertension or hypoxia, and early healthcare requirements.
• This dataset is suitable for evaluating secure Internet of Things (IoT) data-transmission protocols, such as MQTT, using simulated but realistic time-series data streams commonly encountered in IoT systems.
• Monitoring and testing of an autonomous alert system that can notify caregivers or medical services, which has the potential to reduce emergency response time and extreme health outcomes.
• Target users: Researchers in AI and IoT for elderly care, educators, and students prototyping machine learning models.
• Novelty and reusability: Provides a multimodal, labelled dataset for fusion model benchmarking and protocol testing; openly extensible for custom simulations.

## 2 Background

The elderly population poses a significant challenge to health systems worldwide [1,2]. Older adults live alone and have a risk of chronic diseases and falls, which leads to high rates of death and hospitalization [1]. If the above-mentioned accidents occur, intervention at this early stage can avoid severe complications or even death; therefore, cost-effective and non-invasive monitoring devices are required to achieve timely intervention [2]. Traditional monitoring approaches are inadequate for providing continuous real-time monitoring [2,3].

The Internet of Things and Artificial Intelligence are transformative solutions [3,4]. Motor and health movements can be sensed in real time using Internet of Things sensors [3,5], and AI models may learn from these data to recognize anomalies or early ominous events on their own findings [2,4,6]. A major challenge in this domain is the limited availability of large-scale, high-quality labeled datasets for training and cross-validation [[7], [8], [9]]. The present study addresses this gap by providing a realistic, fully synthetic dataset that emulates outputs from a wearable monitoring device to facilitate methodological research. This is crucial because the majority of the available activity datasets are proprietary and hence biased in terms of their replicability and generalization [10].

## 3 Data Description

The dataset simulates recordings from 17 synthetic subjects representing elderly individuals aged 60–70 years, stored in CSV format, with 16 attributes. These attributes include physiological signals (heart rate, SpO₂, and body temperature), motion sensor data from tri-axial accelerometers and gyroscopes, and derived features such as acceleration and gyroscopic magnitudes, as well as heart rate variability. In addition, the dataset contains labeled health conditions and binary indicators of fall events and overall health risk detection.

The dataset contains 612 records simulating 17 subjects (exactly 36 records per subject over approximately 3-hour periods). Data are stored in a single CSV file. Class distributions: health_condition (Normal: 187 records; Hypertension: 85; Hypotension: 85; Fever: 85; Hypoxia: 85; Fall: 85); fall_detected (1: 85; 0: 527); health_risk (1: 425; 0: 187). Timestamps are sequential at 5-minute intervals within each repeated cycle, enabling temporal analysis per subject (segment by timestamp repetition, as no explicit subject ID). The repository includes one CSV (UTF-8 encoded, ∼60 KB) and README.

**Fig. 1:**
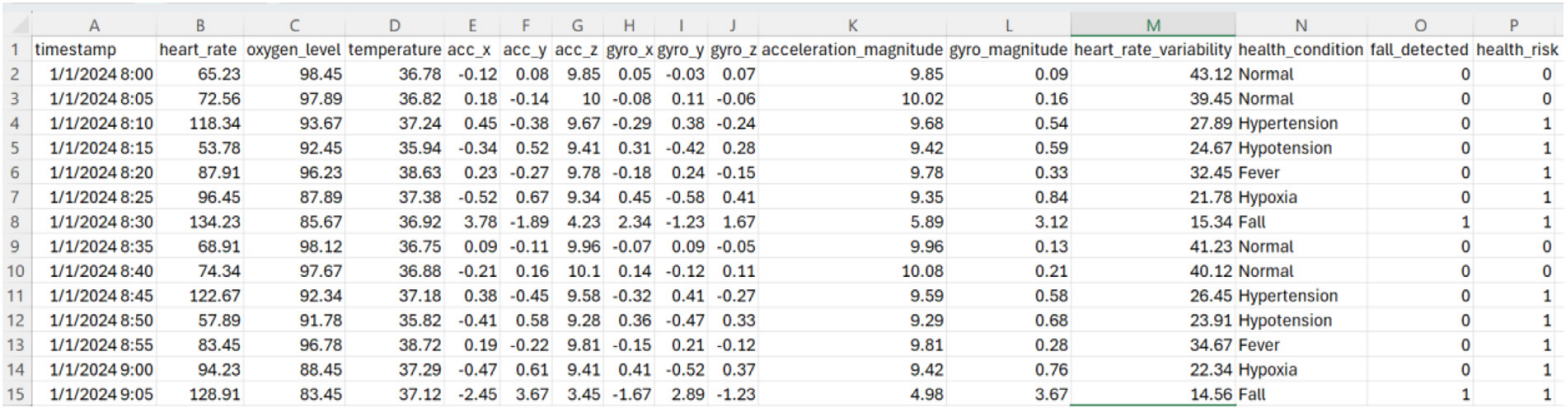
Example of dataset (.csv) open with numbers application.

Table 1 summarizes key physiological and motion-related metrics, including heart rate, SpO₂, body temperature, and accelerometer/gyroscope data, alongside qualitative labels for health status and fall events. The dataset supports machine learning applications for multi-class classification, disease detection, fall recognition, and health risk assessment. This dataset can be used to simulate real-time IoT streams for testing secure protocols such as MQTT and alert systems. By providing structured, realistic, and reproducible data, it facilitates the development of predictive models and smart healthcare solutions that enhance elder safety, proactive care, and overall well-being. An example of the dataset in CSV format is shown in Fig. 1, illustrating the structure and organization of the recorded sensor variables.

**Table 1:** Summary of dataset with descriptions and data types for health monitoring of the elderly.

Attribute	Description	Data Type
timestamp	Date and time of reading (Format: M/D/YYYY H:MM).	Datetime
heart_rate	Heart rate was expressed in beats per minute (BPM).	Float
oxygen_level	Blood oxygen saturation (SpO₂) percentage.	Float
temperature	Body temperature in °C.	Float
acc_x, acc_y, acc_z	Tri-axial acceleration values (m/s²).	Float
gyro_x, gyro_y, gyro_z	Tri-axial angular velocity values (rad/s).	Float
acceleration_magnitude	Magnitude of the acceleration vector: √(acc_x² + acc_y² + acc_z²).	Float
gyro_magnitude	Magnitude of gyroscopic vector: √(gyro_x² + gyro_y² + gyro_z²).	Float
heart_rate_variability	Time variation between heartbeats (ms).	Float
health_condition	Health state labels: Normal, Hypertension, Hypotension, Fever, Hypoxia, Fall.	Categorical
fall_detected	Binary indicator (0 = No fall, 1 = Fall detected).	Integer (0/1)
health_risk	Binary indicator (0 = Normal, 1 = At risk).	Integer (0/1)

## 4 Materials, Methods and Experimental Design

This section outlines the dataset composition along with the data preprocessing steps, model training procedures, and evaluation methods applied to assess the analytical usability of the dataset.

### 4.1 System architecture

**Fig. 2:**
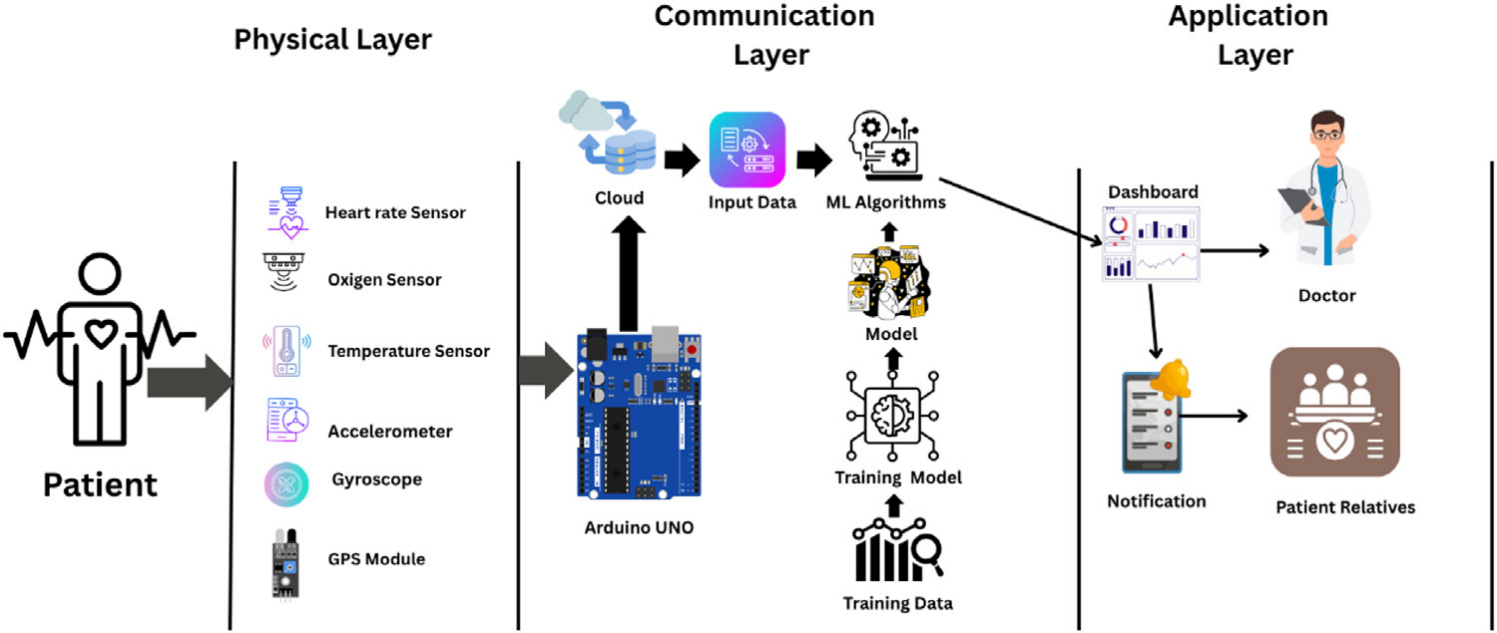
Proposed system architecture of the IoT-based health monitoring system.

The dataset was generated within the context of a conceptual Internet of Things (IoT) based health monitoring architecture developed specifically to simulate realistic data flow in remote elderly care scenarios. This architecture was not implemented as a deployable system; rather, it was designed solely as a structured framework to guide data generation (Fig. 2). The conceptual model is organized into three logical layers to represent end-to-end sensing, transmission, and decision-making processes in a controlled and reproducible manner.

The Physical Layer (Data Acquisition Layer) represents wearable sensing devices that continuously capture multimodal physiological and motion-related signals, including heart rate, blood oxygen saturation (SpO₂), body temperature, tri-axial acceleration, and angular velocity. These signals form the raw inputs used to construct the dataset and emulate continuous monitoring of elderly individuals.

The Communication Layer (Data Transmission and Processing Layer) models the secure transmission of sensor readings to a cloud-based environment using lightweight IoT communication protocols. Within this layer, the data are temporally synchronized, preprocessed, and organized into structured time-series records suitable for storage and downstream analysis.

The Application Layer (Decision and Alert Layer) represents the analytical context in which the dataset may be used, including machine learning–based classification of health conditions and fall events. This layer conceptually supports event detection and alert generation; however, it is included solely to contextualize dataset usage rather than to describe a deployable system.

This layered architecture provides a clear and reproducible context for understanding how the dataset was structured and how it may be reused to evaluate AI-driven health monitoring and fall detection pipelines.

### 4.2 Sensor layer and component specifications

The dataset simulates a wearable sensor layer designed to capture multimodal physiological and motion-related signals relevant to elderly health monitoring and fall detection. This layer provides the foundational inputs used to construct the dataset and reflects sensing configurations commonly reported in IoT-based healthcare research.

The technical characteristics, sensing modalities, and typical applications of the simulated sensors and modules are summarized in Table 2. Detailed hardware-level implementation is intentionally abstracted, as the primary purpose of this section is to document the types of signals represented in the dataset and their relevance for downstream data analysis and machine learning-based evaluation.

**Table 2:** Specifications of wearable sensors and modules used in the elderly health monitoring system.

Component	Type	Technology	Application	Output Type	Common Uses
Heart Rate (HR) Sensor	Biomedical Sensor	Photoplethysmography (PPG)	Measures pulse rate in BPM for cardiovascular monitoring	Analog or Digital	Health monitoring, wearable fitness devices
SpO₂ Sensor	Biomedical Sensor	Optical Sensing (Red and IR LEDs)	Monitors peripheral oxygen saturation (SpO₂) levels	Analog or Digital	Respiratory monitoring, fitness tracking, patient care
Temperature Sensor	Biomedical Sensor	Thermistor / Semiconductor	Detects and monitors body temperature variations	Analog or Digital	Fever detection, continuous temperature monitoring
Accelerometer & Gyroscope	Motion Sensor	MEMS Technology	Detects body movements, orientation, and falls	Digital	Fall detection, activity recognition, motion tracking
GPS Module	Communication Module	Satellite Positioning System	Provides geolocation and tracking during emergency or fall events	Digital	Emergency response systems, location tracking

### 4.3 Data collection, augmentation, curation, and simulation

Continuous multimodal wearable sensing data were synthetically generated to capture fine-grained health dynamics under controlled simulation settings. The simulated data streams included tri-axial accelerometer and gyroscope signals representing posture, activity, and impact patterns; peripheral skin temperature; oxygen saturation (SpO₂); and heart rate signals modeled from photoplethysmography (PPG) or electrocardiography (ECG) waveforms. All channels were time-stamped and synchronized, with IMUs sampled at 50–100 Hz and physiological signals at 0.5–1 Hz. Extracted features comprised Euclidean norms, posture indicators, windowed statistics, and heart rate variability (HRV).

To increase variability without altering labels, training-time augmentation was applied. Physiological signals were perturbed using bounded noise, baseline drift, and mild time warping, while IMU signals underwent three-dimensional rotations, axis-wise jitter, and magnitude scaling. Fall events were diversified through controlled morphology edits, dropout, and quantization to reflect realistic sensor and hardware effects. All perturbations were constrained to clinically plausible ranges.

Data quality was enforced through a staged curation pipeline covering schema validation, physiological plausibility checks, artifact handling, temporal alignment with resampling, event-level de-duplication, and systematic label auditing.

To address class imbalance and rare events, condition-specific priors were used to generate synthetic sequences for Normal, Hypertension, Hypotension, Fever, Hypoxia, and Fall states. Fall events were modeled using a three-phase template consisting of destabilization, impact, and post-impact inactivity or orientation change. Feature extraction and transparent rule-based labeling were applied consistently across all generated samples. Simulation parameters were fixed per release to ensure reproducibility, with detailed distributions summarized in Table 3. Labeling thresholds were aligned with established AHA and WHO guideline criteria [11,12].

**Table 3:** Statistical parameters for normal health_condition state (derived from dataset).

Parameter	Min	Max	Mean	Std
Heart Rate (BPM)	65.23	74.34	70.3	2.9
Oxygen Level (%)	97.45	98.45	98.0	0.3
Temperature (°C)	36.75	36.88	36.81	0.04

### 4.4 Communication layer

The sensor readings were transmitted to a cloud server using the MQTT protocol, which was chosen for its lightweight nature and secure communication, both of which are essential for IoT-based health monitoring. Once received, the data were stored in a structured database and underwent preprocessing steps such as cleaning, normalization, and feature extraction to ensure their reliability and consistency across all samples.

### 4.5 Application layer

Machine learning models deployed on a cloud were used to classify the health status of individuals. Four models, including Support Vector Machine (SVM), Random Forest (RF), Gradient Boosting (GB), and Logistic Regression (LR), were trained on the processed dataset. Each data record was categorized into one of four possible classes:

• Class 0: Normal health, no fall
• Class 1: Abnormal health, no fall
• Class 2: Normal health, fall
• Class 3: Abnormal health, fall

This layer conceptually illustrates how dataset outputs may be used in alert-oriented monitoring scenarios, including alert messages containing key health parameters and GPS coordinates.

### 4.6 Dataset and preprocessing

The dataset, named *healthmonitoringandfalldetection.csv*, contained multimodal sensor readings that included both physiological data (heart rate, SpO₂, and body temperature) and motion data (accelerometer and gyroscope signals). The preprocessing workflow involved several key steps:

• Handling missing values and filtering out noise
• Applying Min–Max normalization to maintain consistency across features
• Conducting correlation-based feature selection to eliminate redundant variables

## 5 Model Training and Evaluation

This systematic process ensured that each model was rigorously evaluated and capable of accurately identifying both health conditions and fall events in real-time. The near-perfect accuracies (e.g., 0.999 for SVM and Random Forest) may stem from the deterministic nature of the simulation rules aligning closely with labeling criteria, potentially reducing classification challenge and leading to optimistic benchmarking. Caution is advised for real-world extrapolation; validate models on empirical data.

To ensure balanced model training and reliable evaluation, the dataset was split into two subsets: 80 % for training and 20 % for testing purposes. To improve model generalization and minimize overfitting, all four machine learning algorithms, including SVM, Random Forest, Gradient Boosting, and Logistic Regression, were trained and validated using a 10-fold cross-validation approach.

### 5.1 Accuracy and F1-score performance

**Fig. 3:**
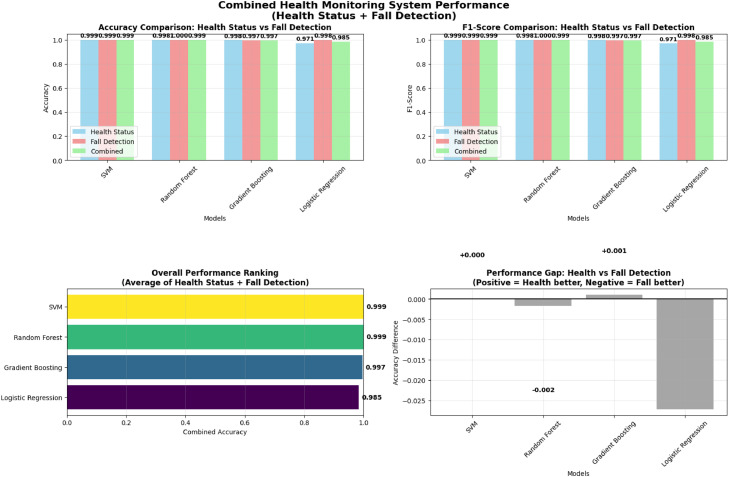
Combined health monitoring system performance (Health Status + Fall Detection).

Fig. 3 presents the accuracy and F1-score obtained by the evaluated classifiers for the combined health condition and fall detection task. Overall, high and consistent performance was observed across all models. Support Vector Machine and Random Forest achieved near-identical accuracy and F1-score values of approximately 0.999, indicating strong and stable classification performance. Gradient Boosting also performed well, with both metrics reaching approximately 0.997. Logistic Regression demonstrated comparatively lower, though still high, performance with accuracy and F1-score values around 0.985.

### 5.2 Overall performance ranking

The lower-left panel of Fig. 3 shows the overall performance ranking, which averages the outcomes of both the health and fall detection tasks. SVM and Random Forest tied for the top spot with a combined accuracy of 0.999, followed by Gradient Boosting at 0.997. Logistic Regression ranked last with 0.985. Performance differences likely reflect differences in model capacity and support comparative benchmarking.

### 5.3 Performance gap analysis

The lower-right panel of Fig. 3 depicts the performance gap analysis, which measures the difference between the accuracy of health monitoring and fall detection (Health Accuracy – Fall Accuracy). SVM maintained a perfect balance with a gap of 0.000, showing equal effectiveness across both tasks. Gradient Boosting exhibited a slight bias toward health monitoring (+0.001), while Random Forest leaned slightly toward fall detection (–0.002). Logistic Regression showed the largest performance gap (–0.027), indicating reduced adaptability to complex multimodal sensor inputs. These gaps illustrate differences in classifier behavior and support comparative benchmarking.

SVM and Random Forest achieved the highest and most balanced results across evaluation metrics. These outcomes indicate that the simulated dataset provides sufficient structural complexity to support benchmarking across diverse classification methods. Logistic Regression showed comparatively lower performance, consistent with the nonlinear characteristics embedded in the dataset features rather than reflecting system-level conclusions.

### 5.4 Confusion matrix analysis

**Fig. 4:**
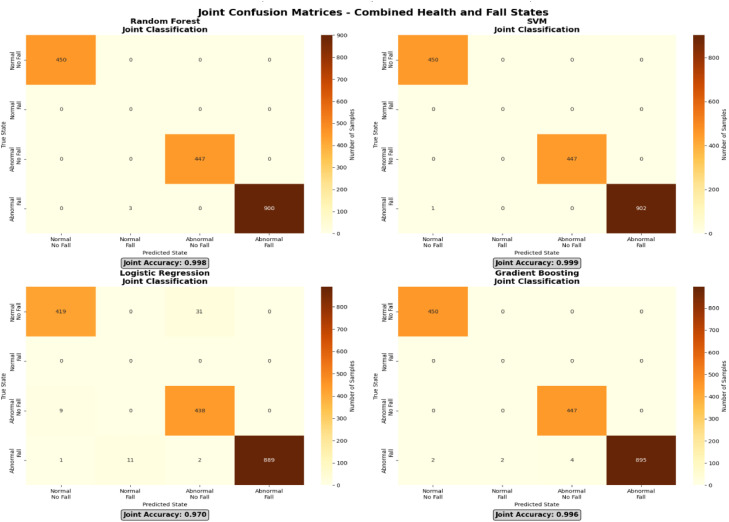
Confusion matrices for combined health and fall states.

The combined confusion matrices for Support Vector Machine (SVM), Random Forest (RF), Gradient Boosting (GB), and Logistic Regression (LR) for joint health status and fall classification are presented in Fig. 4. The matrices illustrate model predictions across four combined states: Normal–No Fall, Normal–Fall, Abnormal–No Fall, and Abnormal–Fall.

Overall, high classification accuracy was observed across all models. SVM and Random Forest achieved near-perfect performance, with accuracy values close to 1.00, followed by Gradient Boosting with slightly lower but still strong performance. Logistic Regression showed comparatively lower accuracy, reflecting its more limited capacity to capture complex, nonlinear relationships in multimodal sensor data. These results further demonstrate the suitability of the dataset for benchmarking multiple classification approaches in combined health condition and fall detection tasks.

## Limitations

The dataset is fully simulated and may not capture the real-world variability, noise, and irregularities found in actual biomedical sensor data, including sensor noise arising from hardware imperfections, inter-subject differences in physiology or behavior (e.g., varying mobility levels or comorbidities), device heterogeneity across manufacturers, and environmental factors such as ambient temperature, humidity, or electromagnetic interference that affect sensor accuracy in elderly populations. All simulated individuals are aged 60–70 years, with no variation in sex, comorbidities (e.g., diabetes or arthritis), mobility levels, or behavioral patterns (e.g., sedentary versus active lifestyles), which restricts generalizability and limits personalization research. The dataset employs an artificially balanced class distribution that does not reflect real-world prevalence, where falls and abnormal health events are relatively rare, and may therefore inflate performance metrics; realistic deployment scenarios would require additional techniques such as class weighting or resampling. While timestamps provide short-term sequential structure (approximately three hours per subject), the dataset does not model long-term continuity, such as multi-day monitoring or disease progression, limiting its suitability for longitudinal analysis. The near-perfect model performance reported may partially result from deterministic simulation rules aligning closely with labeling criteria, leading to optimistic benchmarking outcomes that should be validated against empirical data. Finally, the simulated GPS and communication-related fields are included solely for demonstration of IoT data handling and dataset integration and should not be interpreted as suitable for spatial analysis, real-time deployment, or clinical decision-making. Accordingly, the dataset is explicitly intended for benchmarking and methodological development within controlled research settings, rather than for clinical validation or operational healthcare deployment.

## Ethics Statement

The authors adhered to the ethical standards of Data in Brief and confirmed that this study did not involve human participants, animal experiments, or data collection from social media.

## CRediT Author Statement

**Md. Reazul Islam:** Conceptualization, implementation, Methodology, Result, Writing Original & Editing; **Md. Owafeeuzzaman Patwary:** Literature review, Result, background writing, formating, grammar correction; **S M Ashiqul Islam:** Review and Supervise.

## Data Availability

Mendeley DataHealth monitoring and fall detection (Original data). Mendeley DataRemote Health Monitoring and Fall Detection in Elderly People (Original data).
